# Crosstalk among plant hormone regulates the root development

**DOI:** 10.1080/15592324.2024.2404807

**Published:** 2024-09-16

**Authors:** Yuru Ma, Jiahui Xu, Jiahong Qi, Dan Zhao, Mei Jin, Tuo Wang, Yufeng Yang, Haojia Shi, Lin Guo, Hao Zhang

**Affiliations:** a Ministry of Education Key Laboratory of Molecular and Cellular Biology; Hebei Research Center of the Basic Discipline of Cell Biology, Hebei Collaboration Innovation Center for Cell Signaling and Environmental Adaptation; Hebei Key Laboratory of Molecular and Cellular Biology, College of Life Sciences, Hebei Normal University, Shijiazhuang, China; b College of Life Sciences, Hengshui University, Hengshui, China

**Keywords:** RAM, hormone, auxin, cytokinin, brassinosteroid, crosstalk

## Abstract

The plant root absorbs water and nutrients, anchors the plant in the soil, and promotes plant development. Root is developed from root apical meristem (RAM), which is formed during embryo stage and is maintained by dividing stem cells. Plant hormones have a predominant role in RAM maintenance. This review evaluates the functional crosstalk among three major hormones (auxin, cytokinin, and brassinolide) in RAM development in Arabidopsis, integrating a variety of experimental data into a regulatory network and revealing multiple layers of complexity in the crosstalk among these three hormones. We also discuss possible directions for future research on the roles of hormones in regulating RAM development and maintenance.

## Introduction

In postembryonic development, plant organs form from a small number of stem cells^1,2^ located in stem cell niches (SCNs) in the shoot apical meristem (SAM), root apical meristem (RAM), and vascular cambium (VCAM). The stem cells within these meristems undergo division to generate new tissue types. While some daughter cells differentiate, others retain their pluripotency, ensuring the self-renewal of the stem cell population.^1–3^ The dynamic balance between stem cell division and differentiation maintains meristem function and promotes plant growth and development.^4^

Plant hormones regulate plant development and growth from embryonic development to senescence and death, including regulating RAM development and maintenance. In plants, hormone biosynthesis, transport, and signal transduction establish gradients of hormone accumulation and response, leading to different cell fates.^5,6^ Here, we focus on how plant hormones regulate RAM development, using *Arabidopsis thaliana* as an example to discuss the roles of auxin, cytokinins (CK), and brassinosteroids (BR), and explore the crosstalk among these hormones in RAM development and maintenance.

## Basic structure of the RAM and Root

From the root tip to the top (closest to the shoot), the root is divided into the root cap (a protective layer over the meristem), the meristematic zone, the elongation zone, and the differentiation zone. The meristematic zone in RAM contains the quiescent center (QC) in the middle, the proximal meristem (PM) above, and the distal meristem (DM) below. The QC is surrounded by stem cells to form the SCN in RAM.^2,7^ Signals from the QC maintain the stem cell activity of RAM. These signals involve the WUSCHEL-RELATED HOMEOBOX5 (WOX5) pathway,^8,9^ the SHORT ROOT (SHR) – SCARECROW (SCR) pathway, and the PLETHORA (PLT) pathway, as described in recent review articles.^10,11^ The QC does not usually divide under normal conditions, but it can replenish damaged stem cells by asymmetric division which can produce daughter cells – some to replace the original QC, and some to replace the surrounding stem cell population. The DM comprises only one type of cell: the columella stem cell (CSC, or columella initial, CI). The main components of the root cap are columella cells (COL/CC). In Arabidopsis, division of the CSCs produces four layers of CCs (S1–S4), these layers protect RAM and are involved in sensing gravity signals.^12,13^

The elongation zone is the region of fastest root growth, where the root responds to gravity.^14^ In the differentiation zone, cells differentiate into root cells with specialized functions that form the root tissue (from the outside to the inside) of the epidermis, cortex, endodermis, pericycle, and vasculature. The pericycle and vascular tissues constitute the stele.^15^ In the transition zone (TZ), between the meristematic region and the elongation zone, division stops and cells begin to differentiate.^16^

## Hormonal regulation of RAM development, activity, and maintenance

### Auxin

Auxin is mainly synthesized in the active parts of plants such as young leaves, SAM and RAM, and then transported to other parts.^17^ Auxin plays a key role in regulating RAM development by inducing root initiation, increasing lateral root number and promoting RAM size.^18,19^ Auxin accumulation at the basal end of embryo induces the formation of RAM. The root tip retains an auxin maximum throughout development, which is required for RAM maintenance and proper cell differentiation. Moreover, RAM initiates *de novo* from cells in the pericycle of the main root.^19–22^

WOX5, expressed in the QC, inhibits the differentiation of DSC^9,23^ and influences auxin responses, biosynthesis, and transport in the RAM. Mathematical modeling demonstrated that WOX5 influences the auxin distribution in RAM and WOX5 regulates DM function by adjusting local auxin biosynthesis. *In vivo* and *in silico* works showed that WOX5 activates *TRYPTOPHANAMINOTRANSFERASE ENZYME 1* (*TAA1*) gene expression, which encodes an enzyme included in the beginning steps of auxin biosynthesis, and this activation increases local auxin biosynthesis and auxin levels, resulting in auxin redistribution and changes in CC self-renewal and differentiation. The WOX5–TAA1–auxin circuit is one of the key parts of the complex gene network guiding maintenance of CSCs and CC development.^24^

Auxin transport also affects the auxin distribution in root. In the primary root, the PIN-FORMED (PIN) auxin transporters PIN1, PIN3, and PIN7 mediate auxin flow to the apical regions of root, thus resulting in high auxin concentrations in SCN. *PIN3*, *PIN4*, and *PIN7* are expressed in CCs and the encoded PINs distribute auxin to the root cap and epidermis. Also, apically localized PIN2 mediates auxin upward flow into the elongation zone.^25–27^

Auxin mainly affects root SCN maintenance by inhibiting QC activity and promoting RAM differentiation.^28–30^ Auxin mediates the transcriptional activity of AUXIN RESPONSE FACTORS (ARFs), which interact with APETALA3/ETHYLENE RESPONSE FACTOR (AP3/ERF) and NAC transcription factors to regulate root tip cell division and differentiation.^31^ Among the ARFs family, ARF5 and ARF7 promote the expression of *PLT* genes, which contributes to the differentiation of lateral root cap cells.^3,32^ *ARF10* and *ARF16* have high sequence similarity, and are post-transcriptionally regulated by *miR160*.^30^ Consistent with the phenotype of *miR160* overexpression line, the *arf10arf16* double mutant exhibits retarded root growth, insensitivity to gravity, no accumulation of starch in CCs, and abnormal division and enlargement of the initiating cells in CCs.^28,30^ In addition, AUXIN RESISTANT3 (AXR3) is a member of the Aux/IAA family and inhibits auxin signaling by repressing downstream ARFs. The WOX5–AXR3 feedback loop is crucial for maintaining the auxin signaling gradient in roots and the auxin-mediated differentiation of CSCs.^33^

**Figure 1.**
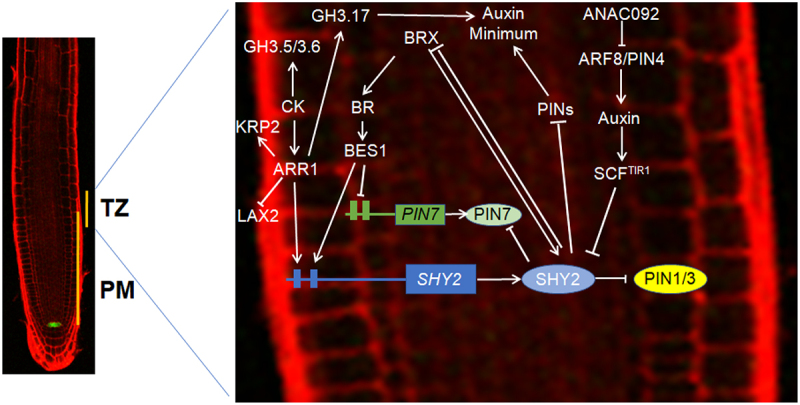
Regulatory network of the hormonal crosstalk involved in regulating RAM function and maintenance.

Besides the main components in auxin biosynthesis, signal transduction, more co-effectors were uncovered to function in RAM maintenance through the auxin pathway. ANAC092^34^ plays a pivotal role in lateral root formation, as evidenced by increased lateral root production in *ANAC092*-overexpressing transgenic plants.^35^ Moreover, ANAC092 exerts control over primary root development. In the *ANAC092*-overexpressing line, there was a notable alteration in RAM length, resulting in shorter primary roots compared to the wild type. Conversely, the *anac092* mutant root was longer than the wild type. Expression of the auxin biosynthetic gene *YUCCA2*, transporters *PINs*, and response factors *ARFs* were downregulated in *ANAC092*-overexpressing transgenic plants. For example, ANAC092 binds to the promoters of *ARF8* and *PIN4*, thereby reducing their promoter activity. These results indicate that ANAC092 negatively regulates root elongation by controlling the auxin pathway^36^ (Figure 1).

Calcineurin B-like proteins (CBLs) and their interacting protein kinases (CALCINEURIN β-LIKE INTERACTING PROTEIN KINASE, CIPKs) constitute signaling modules that relay calcium signals. In plants, CIPK25, a member of the CIPKs family, is involved in regulating RAM size via auxin. Loss-of-function *cipk25* mutant exhibits shorter roots, a slower root growth rate, and fewer RAM cells phenotype. Moreover, *cipk25* mutant line also showed compromised auxin transport and auxin-responsive promoter activity. In Arabidopsis root, reduced expression of auxin-responsive genes including auxin efflux carriers, impaired auxin transport, and low expression of *PINs* were resulted from *cipk25* mutant. CIPK25 was required for optimal expression of certain members of auxin efflux carriers and *ARFs*.^37^ In conclusion, the gradual distribution of auxin in post-embryonic roots promotes QC quiescence and maintains the homeostasis of SCN and the proper differentiation of their daughter cells.

## Cytokinin

CK signal transduction involves a histidine-to-leucine phosphorelay. Three histidine kinases (AHKs), six histidine phosphate transfer factors (AHPs), and 23 ARABIDOPSIS RESPONSE REGULATORs (ARRs) participate in CK signal transduction in Arabidopsis.^38^ Most type-A *ARRs* are primary CK responsive genes, rapidly be induced by CK without requiring *de novo* protein synthesis. Interestingly, multiple type-A *ARR*s binding sites are found in the promoter regions of type-B ARRs.^39,40^ Among the 11 members of the type-B ARRs family, at least 5 members, namely *ARR1*, *ARR2*, *ARR10*, *ARR11* and *ARR12*, are involved in CK signal transduction.^41–43^ Notably, *ARR1*, *ARR10*, and *ARR12* are predominant, actively participating in the response to most CK stimuli. Functional redundancy among family members is evident, as only in multiple mutants can an obvious CK response defect phenotype be observed. CK oxidase (CKX), encoded by a polygenic family, exhibits diverse expression patterns, subcellular localizations and enzymatic characteristics among its members. Studies have shown that CK treatment can rapidly induce the expression of certain *CKX* genes in both Arabidopsis and rice.^44^ Exogenous application of CK can rapidly up-regulate the expression of *CKXs*, while overexpression of *CKXs* will lead to the decrease of endogenous CK content and various developmental defects of plants.^45^

CK promotes growth cessation in the root.^46^ Through time-lapse imaging, genetic studies, and computational analysis, CK has been found to repress root growth in the elongation zone, leading to growth cessation and accelerating the transition of cells from elongation to differentiation.^46^ Arabidopsis roots were examined using confocal laser scanning microscopy after seedlings grew for 5 days post-germination. Analysis of time-lapse images of the root, conducted using MorphoGraphX software, revealed that the cell division rate peaked at 700 μm from the QC, the point that divides the elongation zone into the root elongation zone and the stem elongation zone. Subsequently, the cell division rate gradually decreased toward the upper regions of the root.^46^ The *arr1arr12* double mutant, characterized by a decreased CK signal compared with the wild type, was studied alongside the *phb-1d* mutant, which exhibits enhanced CK biosynthesis. The research findings demonstrated that CK promotes growth arrest during the process of cell differentiation.^46^ Furthermore, osmotic treatments applied to wild-type roots showed that the contraction of cell length in the elongation zone could reflect changes in cell wall hardness. Interestingly, roots treated with CK exhibited a significant reduction in cell contraction toward the stem elongation zone, indicating that CK-mediated cessation of elongation zone growth is associated with cell wall hardening. In addition, auxin is also involved in cell wall hardening and growth arrest.^46^ Using *DR5v2:ntdTomato*, an auxin response reporter, the CK treatment could significantly induce the expression of *DR5v2:ntdTomato* signals in both the main elongation region and differentiation region in the wild type. However, this response was not observed in *aux1-10*, a variant of the auxin transporter AUX1.^46^ These results suggest that the induction at the same position is not significant and CK partially promotes the auxin activity in the stele of the elongation zone through AUX1.^46^ In conclusion, CK plays key roles in promoting root differentiation, accompanying meristem into the TZ of rapid growth, and promoting growth cessation in the root. The QC maintains RAM, ensuring that root growth. PHABULOSA (PHB) is independent of QC controlled root growth. In *shr* mutant plants, PHB is actively restricted by SHORT-ROOTS (SHR) in the meristem to the central region of the stele through *miR165/6*, inhibiting RAM activity, resulting in root growth arrest. The high concentration of PHB in the stele achieves this by regulating B-ARR activity through a independent QC pathway. Remarkably, the RAM activity and growth of *shr phb* double mutant were significantly restored, while the QC was still absent. However, the presence of QC may be required to maintain continuous root growth. SHR maintains root growth through two different pathways: by regulating PHB levels in the root stele, and by maintaining QC identity.^47^

In Arabidopsis RAM, the antagonism of auxin and CK regulates this balance by locating the TZ, where mitotically active cells lose the ability to divide and initiate differentiation programs. In animals, a major regulator of both cell division and cell differentiation is the tumor suppressor protein RETINOBLASTOMA. Similar to homologues in animal systems, the plant RETINOBLASTOMA-RELATED (RBR) protein regulates the differentiation of meristem cells in the TZ by allowing the mRNA accumulation of *ARF19*, a transcription factor involved in cell differentiation. The RBR and CK-dependent transcription factor ARR12 collaborate to activate the transcription of *ARF19*, which is involved in promoting cell differentiation and thus promoting root growth.^48^

The CK response factors (CRFs) are a group of related AP2/ERF transcription factors that are transcriptionally induced by CK. The role of CRF in the growth and development of Arabidopsis by analyzing the lines with decreased and increased CRF function. Although a single *crf* mutation does not have an obvious phenotype, the disruption of multiple CRFs leads to larger garlands, delayed leaf senescence, smaller RAM, reduced primary and lateral root growth, and, in etiolated seedlings, shorter hypocotyls. In contrast, overexpression of CRF typically leads to the opposite phenotype. *crf1,2,5,6* mutants are lethal to embryos, indicating that CRF function is critical for embryonic development. Disruption of CRF results in partial insensitivity to CKs in root elongation assays and affects the basal expression of a large number of cell division protein regulatory genes, including type-A ARRs, while not impairing the induction of cell division peptides by type-A ARRs. In *crf1,3,5,6* mutants, genes encoding homeobox transcription factors are misexpressed, including STIMPY/WOX9, which is essential for maintaining root and shoot apical meristem. Indeed, previous studies have investigated the association of STIMPY/WOX9 with CKs. CRF transcription factors play important roles in multiple aspects of plant growth and development, in part through complex interactions with CK signaling.^49^

Pre mRNA (messenger RNA) splicing is involved in regulating various biological processes in plants. A screen for defects in root growth in Arabidopsis identified an ethyl methane sulfonate mutant defective in pre‐mRNA splicing (*rdm16‐4*). The *rdm16-4* mutant displayed a shortened root phenotype due to fewer cells in the RAM. PLT1 and PLT2 transcription factor genes are crucial for root development. They are alternatively spliced in *rdm16-4* mutants, resulting in the disorder of root SCN and root growth retardation. The root cap of *rdm16-4* contains reduced levels of CK, which can promote the differentiation of developing roots. This reduction is associated with alternative splicing of genes encoding CK signaling factors, such as ARABIDOPSIS HISTIDINE PHOSPHOTRANSFER PROTEIN5 and ARABIDOPSIS RESPONSE REGULATORS (ARR1, ARR2, and ARR11). The expression of the full-length coding sequence of ARR1 or the application of exogenous CK partially rescued the short root phenotype of *rdm16-4*. The alternative splicing of cell division protein signaling components mediated by RDM16 contributes to root growth.^50^

## Brassinosteroids

BRs are a class of polyhydroxyl steroid hormones that promote many aspects of plant growth and development, such as the elongation and thickening of stems and roots, resistance to various environmental stresses, and xylem differentiation.^51,52^ BR regulates root growth in a concentration-dependent manner. Low concentrations of BR promote root growth and inhibit it at high concentration.^53,54^

BR promotes QC cell division through various molecular mechanisms. The BR-INSENSITIVE1 (BRI1)-LIKE 3 (BRL3) signalosome complex containing BRI1-ASSOCIATED RECEPTOR KINASE (BAK1) and BRL1 modulates root growth and development by contributing to provascular and QC cellular activities.^55^ The BR-responsive transcription factors BRS AT VASCULAR AND ORGANIZING CENTRE (BRAVO) and transcription factor ETHYLENE RESPONSE FACTOR 115 (ERF115) regulate QC quiescence. Specifically, BRAVO acts as a cell-specific repressor of QC cell division. BR-REGULATED BRI1-EMS-SUPPRESSOR1(BES1) physically interacts with BRAVO and represses its function. Furthermore, BR can drive QC proliferation by stimulating *ERF115* expression, which induces QC cell division by acting through PHYTOSULFOKINES 5 (PSK5) signaling.^56–58^

BR signaling mediates UPBEAT1 (UPB1)-regulated RAM development.^59^ UPB1, as a bHLH subfamily 14 transcription factor, shows less homology to other members in the bHLH subfamily 14 subfamily and modulates the balance between cell proliferation and differentiation by directly controlling the expression of peroxidases genes in RAM.^60^ BRASSINOSTEROID INSENSITIVE 2 (BIN2), a critical protein kinase in BR pathway, enhances the negative effect of BR signal on RAM development by directly interacting with and phosphorylating UPB1.^59^ Genetic experiments show that UPB1 functions downstream of BR signal transduction. The authors also found that PACLOBUTRAZOL-RESISTANT PROTEIN FAMILY 2/3 (PRE2/3), encoded a basic helix/loop/helix transcription factor that acts downstream of ARF5 in root initiation, could interact with UPB1 and affect the formation of RAM.^59,61^ Therefore, the results reveal new insights into the molecular mechanism of the relationship between BR signal and RAM development.^59^

Meanwhile, BR regulates cell division and differentiation in RAM. Mutants with increased BR signaling or plants treated with BR showed premature differentiation of meristem cells, decreased RAM size, and less overall root growth.^62^ Interestingly, BR regulates the expression of genes, such as *SCR* and *WOX5*, to maintain SCN identity and structure.^63^ BR induced the accumulation of BRASSINAZOLE RESISTANT 1(BZR1) in roots. BZR1, a homolog of BES1, promotes cell division in QC, but suppresses CSC differentiation, opposite to the action of BES1.^64^ Recent study showed that BR and the BZR1-mediated signaling pathway alter the expression/subcellular distribution of PINs, which may change auxin movement. This alteration in auxin accumulation, coupled with the regulation of genes, such as *BRAVO*, *ERF115*, and other root-regulatory genes, may lead to ectopic activation of QC cell division and suppression of CSC differentiation^58,64^ (Figure 1).

The transcription factor BRI1-EMS-SUPRESSOR 1 (BES1) is a master regulator of BR-regulated gene expression. BES1, together with BZR1, drives activated or repressed expression of several genes, and have a prominent role in negative regulation of BR synthesis. Here, we report that the interaction of BES1 with TOPLESS (TPL) through its ERF-associated amphiphilic repression (EAR) motif is critical for the BES1 mediated control of meristem organ boundary formation and the regulation of QC cell division in roots. We demonstrate that TPL binds to the promoters of CUC3 and BRAVO targets through BES1 and inhibits their expression. Ectopic expression of TPL resulted in organ boundary defects and altered QC cell division rate similar to the *bes1-d* mutation, while the *bes1-d* defect was inhibited by the dominant interfering protein encoded by *tpl-1*, and these effects were associated with changes in CUC3 and BRAVO expression, respectively. The critical role of vasopressin TPL in stem and root meristem, which depends on its interaction with BES1 and the regulation of BES1 target gene expression.^65^

CYCP3;1, a P‐type cyclin in root, is specifically expressed in the epidermis of RAM and lateral root crown and can regulate meristem cell division. Mitotic analysis and biochemical studies showed that CYCP3;1 promotes G2-M phase cell division by binding and activating cyclin‐dependent kinase B2‐1 (CDKB2;1). In addition, we found that CYCP3;1 expression is inhibited by BR signaling through BES1, a positive downstream transcription factor in the BR signaling pathway.^66^

In *bri1 brl1 brl3* mutants, protophloem sieve element differentiation is indeed impaired, although this effect may not be mediated by typical downstream BR signaling components. Their small meristem size was entirely caused by reduced cell elongation, accompanied by additional formative cell division in the radial dimension. The overall short root phenotype of BR signaling mutations may be explained by the necessity to adapt to additional formative division, reduced cell expansion, and growth retardation. Tissue-specific re-addition of BRI1 activity partially rescued these defective subpopulations through partial cell autonomous and partial non-cell-autonomous effects. However, protoderm-specific BRI1 expression substantially rescued all major *bri1 brl1 brl3* RAM phenotypes. BR perception in the protoderm is sufficient to systematically transmit BR effects in RAM.^67^

## Crosstalk between auxin and CK

The plant hormones CK and auxin regulate a variety of plant processes and often collaborate to control growth and development. Although much is known about how these hormones act alone, we are only beginning to understand how these signals interact to achieve a comprehensive response. Previous studies have shown that exogenous CK have an effect on the transcription of several PIN efflux vectors. The disruption of type-A ARR, a negative regulator of CK signaling, alters the level of PIN protein and leads to increased sensitivity to the polar auxin transport inhibitor *N*-1-naphthylphthalamic acid. Disruption of 8 of 10 type-A ARR genes affects root development by changing the size of the meristem. The effect of CK on PIN abundance mainly occurs at the post-transcriptional level. Changes in PIN levels in type-A ARR mutants lead to alterations in the distribution of apical auxin measured by *DR5:GFP* reporter, as well as altered patterns of cell division and differentiation in the SCN in meristem. CK, acting through the type-A ARRs, alters the level of several PIN efflux carriers, and thus regulates the distribution of auxin within the root tip.^68^

CK regulates the root elongation through ethylene signaling, while the effect of cell division proteins on RAM size is mediated by ethylene-independent transport. Both exogenous or endogenous modifications of CK levels and cell division protein signals lead to specific changes in the transcription of several auxin efflux carrier genes from the PIN family, which have a direct impact on auxin efflux and auxin distribution in root tips of cultured cells. A new model for the role of CK in regulating root growth: cell division proteins affect auxin transport between cells by changing the expression of several auxin transport components, thereby regulating the auxin distribution, which is very important for regulating the activity and size of RAM.^69^

Auxin interacts with CK to determine the boundary between cell division and differentiation in the RAM.^16^ At the boundary of TZ, cell division stops, and cells begin to elongate and differentiate; further up the root, the cells elongate more (in the elongation zone) and complete their differentiation (in the differentiation zone). The coordinated activities in these areas and the maintenance of TZ position stabilize the size of the RAM and ensure the continuous growth of roots.^70^ The CK-responsive transcription factor ARR1 inhibits *PIN* expression in the TZ by directly activating the transcription of the auxin signal inhibitor *Aux/IAA SHORT HYPOCOTYL 2*(*SHY2*).^16^ By contrast, auxin activates SHY2 degradation to maintain PIN activity.^16^

The auxin-inactivating gene *GRETCHEN HAGEN 3.17*(*GH3.17*), which encodes an indole-3-acetic acid amino synthase that irreversibly conjugates auxin to aspartic acid or other amino acids, was specifically expressed in CCs, the outermost layer of the lateral root cap (LRC), and differentiated epidermal cells. This expression pattern is consistent with the direct role of ARR1 in controlling *GH3.17* transcription, as *ARR1* is also expressed in the LRC and differentiated epidermis. CK maintains the position of the TZ and controls polar auxin transport and auxin inactivation. The CK-dependent transcription factor ARR1 directly controls auxin inactivation in external tissues by inducing the expression of *GH3.17*. The control of auxin transport and inactivation produces an auxin minimum in the TZ, precisely at the transition from cell division to differentiation^70^ (Figure 1).

In developing roots, cell division decreases and TZ forms after seed germination in the early stages of SCN activation. The decrease of PLT levels triggers cell differentiation to establish the TZ and activate ARR12 to counteract PLT1/2. ARR12 and the PLTs inhibit each other, limiting *PLTs* expression to the SCN, and limiting ARR12 accumulation shootward of the TZ. At slightly later stages, SHY2- and GH3.17-dependent ARR12 antagonizes auxin thus affecting PLT levels and further promoting TZ formation. ARR1, but not ARR12, directly activates *KRP2*, inhibiting cell division. This inhibition allows ARR1 to limit the division-dependent expansion of the PLT gradient by stabilizing the meristem size^58,71^ (Figure 1).

CK controls QC cell division by decreasing the expression of *SCR*, *WOX5*, and the auxin influx carrier genes *AUXIN TRANSPORTER PROTEIN 1* (*AUX1*) and *LIKE AUX 2* (*LAX2*). CK decreases *LAX2* expression via ARR1 and ARR12. ARR1 directly binds to the *LAX2 in vivo*, which suggests that B-type ARRs directly regulate the genes repressed by CK. Loss-of-function mutants of *LAX2* exhibited phenotypes resembling the response to CK treatment, including increased cell division in QC and decreased expressions of *WOX5* and the auxin response reporter *DR5*. CK thus regulates auxin distribution in the RAM and mitotic activity in QC by PINs and LAX2^72^ (Figure 1).

CK regulates RAM development through the auxin pathway^16,70,73,74^ by inducing expressions of *GH3.5* and *GH3.6*. GH3.5 and GH3.6 are involved in regulating RAM activity and size, together with GH3.17.^74^ Compared with wild type, RAM sizes of *gh3.5-1* and *gh3.6-1* were increased, but showed a slight decrease after treatment with CK. CK thus regulates RAM size by activating the expression of *GH3.5* and *GH3.6*^74^ (Figure 1).

Auxin – CK crosstalk also plays a key role for root development in responses to environmental conditions. For example, the essential micronutrient boron (B) causes adverse effects on plant growth and development when present at high levels. 26S proteasome (26SP) is required to maintain the morphology of RAM by modulating the auxin and CK responses under high-B stress. High-B treatment reduced RAM size by inhibiting cell division.^75,76^ REGULATORY PARTICLE AAA-ATPASE 5a (RPT5a) of the 26SP affects high-B-dependent maintenance of RAM size and the *rpt5a* mutant is hypersensitive to high-B stress, showing severe defects in RAM morphology, including disordered cell arrangement around SCN. High-B stress-induced *RPT5a* expression in the entire RAM, accompanied by a strong expression in the stele. The 26SP adjusts the activity of TIR1/AFB-dependent auxin signaling to the appropriate level required for the maintenance of SCN, especially under high-B conditions. The 26SP also regulates CK responses through ARR1 and ARR5,^77^ which may repress auxin responses and be crucial for stem cell proliferation and the size of the stele.^78^

Genetic experiments indicate that there is an antagonistic relationship between SHY2 and CIPK25. For example, CK treatment decreased the expression of *CIPK25* in an ARR1/12-dependent manner. *SHY2* is highly expressed in *cipk25* mutants and the *cipk25* short-root phenotype is completely rescued in the *cipk25 shy2-24* double mutant, similar to *PIN1*, *PIN2* and *PIN3*. CK treatment resulted in a further reduction of *PIN1* expression and meristem size in *cipk25* mutants, suggesting that CK also plays a role in a CIPK25-independent pathway. These observations show that CIPK25 plays a pivotal role in balancing auxin and CK signals in root development,^37^ uncovering a new mechanism whereby CK induces auxin inactivation in the stele to control RAM size (Figure 1).

## Crosstalk between auxin and BR

Auxin interacts with BR to modulate RAM size and activity. The transcription factor BREVIS RADIX (BRX) controls cell proliferation and root elongation by mediating BR and auxin signaling. *BRX*-deficient lines showed a short-root phenotype, with short and CK-insensitive RAMs. Auxin strongly induces the expression of *BRX* and BRX, in turn, promotes BR synthesis, thus enhancing BR signaling and maintaining homeostasis of RAM. Auxin affects *BRX* expression via SHY2, which limits *BRX* expression, resulting in a reduced RAM, while correct *SHY2* expression requires active *BRX*.^6,79–81^

The interaction between BR and auxin in TZ promotes RAM development. The expression of *PIN3* was decreased significantly in the TZ of *brx* mutants, indicating that BRX is a positive regulator of *PIN3* and thus affects auxin transport. BR increased the size of the RAM by upregulating *PIN7* expression and downregulating *SHY2* expression. BES1 directly binds to the promoter regions of *PIN7* and *SHY2*, suggesting that *PIN7* and *SHY2* mediate BR-induced RAM growth as direct targets of BES1. The *shy2* mutants carrying a *PIN7* overexpression construct were sensitive to BR and showed partial inhibition of the short-root phenotype of BR signal deficient mutants. Intriguingly, BR inhibited the accumulation of SHY2 protein in response to CK. These observations show that the crosstalk among BR, auxin and CK regulates root growth through complex network regulation^58,82^ (Figure 1).

The opposite pattern and antagonism of BR and auxin to maintain stem cell balance and optimal root growth. There were low levels of nuclear localized BR activated transcription factor BZR1 in the SCN, while there were high levels of BZR1 in the transition extension region. This BZR1 pattern requires local BR catabolism and auxin synthesis as well as BR signaling. Cell type specific expression of the constitutively active form of bzr1 confirmed that high and low levels of BZR1 are required for normal cell behavior in the elongation zone and QC, respectively. The comparison among BR reactivity, BZR1 targeting, auxin responsiveness and developmental region-specific transcriptome showed that BZR1 mainly activated its target genes expressed in the transition extension region, but inhibited genes in QC and surrounding stem cells. BR and auxin exert opposing effects on gene expression overall. Genetic and physiological interactions support the idea that a balance between the antagonistic effects of BR and auxin is required for optimal root growth. The level and output specificity of BR signals are spatially patterned, in contrast to their synergistic effect in buds. BR and auxin interact in roots to control the spatiotemporal balance of stem cell dynamics required for optimal root growth.^83^

## Crosstalk between Auxin and other plant hormones

Environmental conditions such as drought stress activate abscisic acid (ABA) signaling, which interacts with auxin to regulate RAM activity. AUXIN RESPONSE FACTOR 2 (ARF2)^84^ is a negative regulator of ABA responses and seed germination. The primary root growth of *arf2* mutants are highly sensitive to ABA.^18^ ABA treatment reduces the expression of the auxin efflux carriers *PIN1*, *PIN3*, *PIN4* and *PIN7* in RAM of *arf2-101* mutants. In addition, the *arf2-101 pin1* and *arf2-101 pin4* double mutants were less able to suppress ABA-induced RAM activity compared with the *arf2-101* single mutants. ARF2 induces *PLT1* transcription in RAM and negatively regulates PLT2 protein level. In the dexamethasone (DEX)-inducible transgenic-line *pro35S:PLT2-GR*, PLT2 significantly promoted cell division in the *arf2-101* RAM after induction by DEX and completely inhibited cell differentiation, which could be partially reversed by ABA treatment. This indicates that ABA regulates RAM activity in ARF2-dependent and -independent pathways. ARF2 affects PLTs and PINs to coordinate ABA-mediated regulation of RAM activity.^18,58^

Salicylic acid (SA) is integral to plant responses to pathogen infection, which may affect root growth via growth – defense trade-offs. Exogenous SA regulates the root length of Arabidopsis^85,86^ and specifically induces root waving in a dose-dependent manner.^86^ In addition, high-concentration SA inhibits primary root growth and lateral root development. Low-concentration SA promotes adventitious roots and alters the architecture of RAM. Treatment with exogenous SA leads to changes in auxin synthesis and transport. SA-induced auxin accumulation leads to the formation of more layers of columella initials, an additional cortical cell layer (middle cortex), and extra files of epidermis, cortex, and endodermis cells. Suppression of short root and activation of *CYCLIN D6;1*(*CYCD6;1*) mediated the changes in the radial architecture of the root. These indicate at a low concentration, SA plays a crucial role in shaping RAM structure and root system architecture through the auxin pathway.^87^

Strigolactones (SLs) promote primary root elongation but suppress lateral roots. Recent findings showed that the *shy2* mutant was less sensitive to SLs in the number of RAM cells, and *shy2-31* and *max2* were not sensitive to CK. Therefore, SLs induce SHY2 activity and regulate root tip auxin flux, thereby regulating RAM size.^58,88^

## Conclusions and perspectives

The root system is essential for plant growth, development and responses to the environment.^89^ In plant growth and development, the formation of various tissues and organs depends on meristem activity. RAM is crucial for plant root morphogenesis and the formation of the various root organs. Meristem homeostasis is regulated by a complex network of factors and hormones, which modulate gene expressions to balance cell division and cell differentiation. The interactions among hormones play an important role in the temporal and spatial coordination of root development. The results described above show that the classical plant hormones, auxin, CK, and BR are involved in post-embryonic root organogenesis and interact to regulate RAM formation and maintenance.^90,91^ Indeed, CK and auxin exhibit antagonistic interactions in regulating root development. CK negatively regulates the expression of *PIN1* and *PIN4*, positively regulates the expression of *PIN7*, thus regulating the distribution of auxin and RAM size.^82^ Nevertheless, additional work will be required to fully elucidate the complex mechanisms by which hormone interactions regulate root development.

Cell proliferation and differentiation in the RAM are balanced by complex genetic regulatory networks,^10,92^ in which PLTs form a concentration gradient at the root tip, and redundantly control RAM maintenance in a dose-dependent manner.^3,32,93^ Auxin promotes the expression of genes encoding PLTs, and the establishment of the PLTs concentration gradients are partially independent of auxin.^3,94^ Previous studies have shown that PLTs maintain auxin concentration gradient by regulating *PIN* expression.^95–97^ Given the crosstalk between auxin and other phytohormones discussed above, how PLTs concentration gradients are established and maintained in response to the crosstalk among phytohormones needs to be further investigated.

Technological advances will enable new approaches to elucidate these mechanisms. For example, live cell imaging^98^ now allows us to track multiple molecular markers at the same time for a long time and observe the dynamic changes of molecules in RAM. Computational modeling (Bartocci and Lió, 2016) can simulate and predict the crosstalk between hormones. RNA single-cell sequencing^99,100^ and gene editing technology^101^ will also help us decipher the mechanisms by which hormones regulate root development.

The study of root development helps us understand how plants survive adverse environmental conditions. For instance, root traits can enhance resistance to diseases and insect pests, improve competitive growth, and enable plants to obtain more water and nutrients in conditions where these inputs are limited.^102^ In future research, studies of the adaptive growth mechanism of plant roots at the cytological and molecular level, with the aim of altering root configurations, will improve crop adaptation to special growth conditions, thereby increasing yield.^103^
